# Modification of Physico-Chemical Properties of Acryl-Coated Polypropylene Foils for Food Packaging by Reactive Particles from Oxygen Plasma

**DOI:** 10.3390/ma11030372

**Published:** 2018-03-03

**Authors:** Tomislava Vukušić, Alenka Vesel, Matej Holc, Mario Ščetar, Anet Režek Jambrak, Miran Mozetič

**Affiliations:** 1 Department of Food Engineering, University of Zagreb, Pierottijeva 6, 10000 Zagreb, Croatia; tvukusic@pbf.hr (T.V.); mscetar@pbf.hr (M.Š.); arezek@pbf.hr (A.R.J.); 2 Department of Surface Engineering, Jozef Stefan Institute, Jamova cesta 39, 1000 Ljubljana, Slovenia; alenka.vesel@guest.arnes.si; 3 Jozef Stefan International Postgraduate School, Jamova cesta 39, 1000 Ljubljana, Slovenia; matej.holz@ijs.si

**Keywords:** plasma surface modification, polymer polypropylene, neutral oxygen atom density, initial surface functionalization, food packaging, wettability

## Abstract

This investigation was focused on the influence of long-living neutral reactive oxygen species on the physico-chemical properties of acryl-coated polypropylene foils for food packaging. Reactive species were formed by passing molecular oxygen through a microwave discharge and leaking it to a processing chamber of a volume of 30 L, which was pumped by a rotary pump. The density of neutral O-atoms in the chamber was tuned by adjustment of both the effective pumping speed and the oxygen leak rate. The O-atom density was measured with a catalytic probe and was between 3 × 10^18^ and 5 × 10^19^ m^−3^. Commercial foils of biaxially oriented polypropylene (BOPP) coated with acrylic/ poly(vinylidene chloride) (AcPVDC) were mounted in the chamber and treated at room temperature by O atoms at various conditions, with the fluence between 1 × 10^21^ and 3 × 10^24^ m^−2^. The evolution of the surface wettability versus the fluence was determined by water contact angle (WCA) measurements, the formation of functional groups by X-ray photoelectron spectroscopy (XPS), and the morphology by atomic force microscopy (AFM). The WCA dropped from the initial 75° to approximately 40° after the fluence of a few 10^22^ m^−2^ and remained unchanged thereafter, except for fluences above 10^24^ m^−2^, where the WCA dropped to approximately 30°. XPS and AFM results allowed for drawing correlations between the wettability, surface composition, and morphology.

## 1. Introduction

Today, food technology is constantly evolving in response to different challenges. The changes in consumer demands and the necessity for the production of safe and high-quality foods are responsible for the innovation and improvement of already established food processes. In this sense, the introduction of new technologies could lead to a reduction of the processing time or an improvement in operating conditions, thereby decreasing both environmental and financial costs. Plasma treatments cause several chemical and physical changes on the plasma-polymer interface, which improve the surface properties [1,2,3,4,5,6]. Plasma-induced effects on the polymer surface are nowadays exploited in surface functionalization of the packaging polymers for promoting adhesion or sometimes anti-adhesion [7], enhanced printability [8], sealability [9], assuring anti-mist properties, improving the polymer’s resistance to mechanical failure [1], and adhesion of antibacterial coatings [10,11,12,13,14].

Polypropylene (PP) is an important commercial polymer which is often used for producing package films [15], because of its low cost and good thermal stability. Extruded PP film is amorphous, while the crystallization can be achieved by two-way stretching (monoaxially or biaxially orientated films) at elevated temperatures. Biaxial orientation (BO) slightly improves the silky structure of the film and significantly reduces turbidity, enhancing the barrier properties and flexural toughness at a low temperature. Biaxially oriented polypropylene (BOPP) film is often coated with an additional polymer layer to improve its mechanical properties or barrier properties against gases and moisture. If BOPP is used for food packaging applications, it is often coated with an acrylic layer or poly(vinylidene chloride) (PVDC). Acrylic coating (Ac) is durable, flexible, and resistant to degradation caused by ultraviolet rays [16]. If the PP foil is coated with a PVDC layer, this topcoat enhances PP barrier properties against water vapor and gases. Excellent protective properties of this layer make PP foil suitable for the packaging of confectionery products which require barrier protection from moisture [17].

As mentioned above, plasma can improve surface properties of polymers such as wettability and surface functionalization, and consequently also adhesion properties. This may be important for coating the food-packaging foils with antibacterial layers [18]. Many authors have investigated treatment of a pure PP foil rather than industrial-grade foils, which are often covered with an ultra-thin film of a protective coating. A reason for this might be unknown details regarding the composition and structure of the coating, let alone the method applied for deposition of the coating. Pandirayaj et al. [19] used a low pressure weakly ionized plasma created by a DC glow discharge to improve the wettability of the PP foil. The water contact angle dropped from the original value of 98° down to 58° upon treatment for 10 min. Similar results were reported by Choi et al. [20], who obtained 60° in low-pressure oxygen DC plasma. Additionally, Morent et al. [21] obtained a water contact angle of 60°, although he used an extremely weak plasma at the discharge power of solely 1.4 W and moderate pressure of 5 kPa. A dielectric barrier discharge (DBD) was applied. Leroux et al. [22] obtained the contact angle of 64° using plasma created in air at atmospheric pressure by classical DBD and a treatment speed of 2 m/min. Lower water contact angles were observed by some other authors. Aguiar et al. [23] achieved a water contact angle of 40° (initially 110°) on PP surface treated in oxygen plasma at 700 W and 6.7 Pa. Vishnuvarthanan et al. [24] observed that the contact angle depended on the discharge power (7.2–29.6 W) and treatment time (0–300 s). The lowest water contact angle was ~44°; however, the initial angle was just 74.5°, which indicates that the initial surface was probably already contaminated with surface impurities. Mirabedini et al. [25] obtained a minimal contact angle of 34.4° in RF oxygen plasma at 50 W and 0.35 × 10^5^ Pa. However, Wanke et al. [26] managed to achieve only 24° (initially 98°) at 15 min of treatment. Unlike other authors, who observed a decreasing water contact angle with increasing treatment time until reaching saturation, he observed that at long treatment times (after 15 min of treatment), the contact angle increased to 53°. The reason that some authors obtained such low contact angles can be associated with polymer overtreatment leading to the formation of low-molecular weight fragments (LMWOM) because of polymer degradation [27]. The main papers and key results are summarized in Table 1.

Although oxygen plasma treatment causes beneficial effects such as improved wettability, it also causes other modifications of the surface and sub-surface layer which may not be tolerated. Oxygen plasma is rich in different reactive species and represents a source of ultraviolet (UV) radiation. The reactive gaseous species that interact with a polymer sample include positively charged molecular and atomic ions, neutral atoms in the ground and metastable states, and neutral molecules in both “a” and “b” metastable states and ozone. The major UV radiation occurs at the wavelength of 130 nm due to the transition from a highly excited 2s^2^2p^3^(^4^S°)3s^3^S° state to the ground state (2s^2^2p^4 3^P). The photon energy for this transition is 9.52 eV. The penetration depth of such UV radiation in a polymer material is around a micrometer [28]. The energetic photons cause bond scission and thus modification of the polymer properties well below its surface. Furthermore, there is always some water vapor in a low-pressure plasma reactor. The vapor is the major constitute of the residual atmosphere and is also formed due to chemical etching of the polymer upon oxygen plasma treatment. The water molecules dissociate under plasma conditions and the resulting OH and H radicals are excited upon inelastic collisions with energetic electrons. The excited states de-excite to the corresponding ground states by ration in the UV range: Lyman hydrogen series in the vacuum UV range and OH band of bandhead at 309 nm. All this radiation causes bond scission in the polymer film of a thickness of the order of several µm. The reactive species interact with dangling bonds on the polymer surface, causing the formation of LMWOM that are often volatile. Therefore, rather extensive etching is observed upon the treatment of a polymer material with oxygen plasma [29]. In fact, precise measurements of the oxidation rate for the same polymer exposed to oxygen plasma and only neutral O-atoms at the same O-atom flux on the sample surface showed a two orders of magnitude higher etching rate for the case where synergistic effects of radiation and reactive species were effective [30,31]. Such synergies should therefore be avoided if functionalization of the polymer surface with oxygen functional groups is the goal.

The aim of this research was to examine the effect of surface oxidation of commercial PP foils used for food packaging. Such foils are covered with a very thin acrylic coating. Unlike other authors, neutral reactive particles from late afterglow were used instead of gaseous plasma, because glowing plasma always causes the etching of polymers and the acrylic coating could have been removed by direct exposure to oxygen plasma [30]. Furthermore, in afterglow, a density of oxygen species interacting with the polymer can also be precisely determined. This allowed determination of the minimal oxygen atom fluence necessary for saturation of the surface with polar functional groups and thus optimal wettability at a minimal treatment time.

## 2. Materials and Methods 

### 2.1. Materials

Biaxially oriented polypropylene (PP) films (Bicor 32MB777, ExxonMobil, Antwerp, Belgium) were used in the experiments. One side of the film had an acrylic acid coating and the other side was coated with a thin film of poly(vinylidene chloride) (PVDC), which means that plasma interacted with the coating and not with the PP substrate. Only the acrylic side was treated with plasma. The thickness of the foil was 32 µm.

### 2.2. Plasma Afterglow Treatment

**Figure 1:**
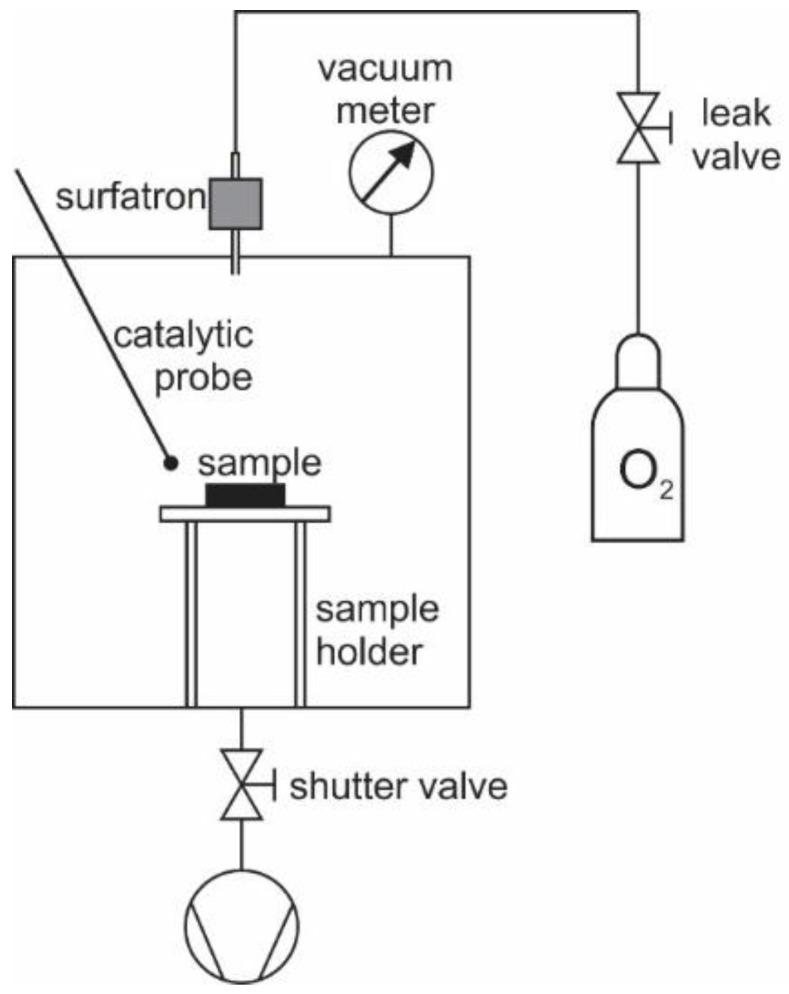
Experimental plasma system used for treating polymer samples.

The polymer foil was cut into pieces of 2 × 2 cm^2^ and treated with reactive neutral oxygen species created in the center of the processing chamber. Oxygen species which were created in the surfatron plasma were passed through the narrow glass tube to the processing chamber. The experimental system is shown schematically in Figure 1. The processing chamber was a pyrex cylinder with a diameter of 33 cm and a length of 40 cm. The chamber was pumped with a two-stage oil rotary pump of a nominal pumping speed of 40 m^3^·h^−1^ and ultimate pressure well below 1 Pa. A zeolite trap was used to prevent back-diffusion of the oil vapor. The pump was mounted on the flange at the bottom of the processing chamber via bellows of a large conductivity at the pressure of 20 Pa and above, and a manually adjustable shutter valve which allowed for suppressing the effective pumping speed in a gradual manner from the maximal speed (40 m^3^·h^–1^) down to zero. The upper flange of the Pyrex tube was equipped with a pressure gauge, a discharge tube, and a movable catalytic probe which was used for O-atom density measurements [36]. Oxygen of commercial purity 99.99% was leaked continuously in the discharge tube through a manually adjusted leak valve. A standard quartz tube with an inner diameter of 6 mm was used. The pressure was measured with an absolute gauge (baratron) calibrated for the pressure range 0.1–100 Pa. A microwave cavity of approximately 5 cm in length was mounted onto the discharge tube and connected to the microwave power supply. The configuration allowed for sustaining the gaseous plasma in the surfatron mode inside the discharge tube. The microwave power was set to 200 W. Continuous leakage of oxygen on one side and pumping of the processing chamber on the other side allowed for a drift of gas through the discharge into the processing chamber. Molecular oxygen from the flask partially ionized, dissociated, and excited in the plasma within the microwave cavity. Charged particles quickly neutralized and excited species relaxed on the way between the gaseous plasma and the processing chamber. Therefore, the only highly reactive oxygen species left for treatment of the polymer samples was neutral O atoms. The density of O atoms above the surface of the polymer samples was measured with a calibrated catalytic probe. The probe consists of a catalytic tip which is heated in the plasma because of the recombination of O atoms to O_2_ molecules on the surface of the catalyst [36]. The temperature of the catalyst is measured by a thermocouple. The heating rate of the probe is proportional to the flux of oxygen atoms. The O-atom density (*n*) was calculated from the probe temperature derivate using the following equation [37]:

(1) $$n=\frac{8\cdot m\cdot c_{p}}{v\cdot W_{D}\cdot \gamma \cdot A}\cdot \left(\frac{dT}{dt}\right)$$

where *m* is the mass of the probe tip, *c*_p_ is its specific heat capacity, *W*_D_ is the dissociation energy of an oxygen molecule, *γ* is the recombination coefficient for O atoms on the catalyst surface, *A* is the area of the catalyst, and d*T*/d*t* is the time derivative of the probe temperature just after turning off the discharge. More details regarding the O-atom density calculation are explained in the works [36,37]. We have used cobalt as the catalyst. This material is particularly suitable for the detection of atomic oxygen at a low density. The lower detection limit of the probe was approximately 2 × 10^18^ at a pressure above 10 Pa, whereas the upper at 10^22^ m^−3^.

The experiments presented here were performed at the pressure of 20 Pa. At these conditions, the O-atom density in the system was 5.3 × 10^19^ m^–3^ when the shutter valve was fully open (the effective pumping speed was equal to the nominal pumping speed of the vacuum pump). By adjusting the shutter and leak valves simultaneously, it was possible to keep the pressure in the processing chamber constant but the O-atom density variable: less opened valves caused a lower atom density because the drift velocity of the gas through the discharge chamber was suppressed by closing valves. A detailed description of this effect was reported elsewhere [38]. Four adjustments of the O-atom density in the vicinity of the samples were chosen: 5.3 × 10^19^, 2.9 × 10^19^, 1.0 × 10^19^, 8.7 × 10^18^, and 3×10^18^ m^−3^. The corresponding fluxes of O-atoms onto the sample surface were calculated as:

*j* = ¼ *nv* (2)

where *n* is the measured density of oxygen atoms and *v* is an average thermal velocity of O atoms at room temperature (*v* = 630 m·s^–1^). The fluence of O atoms to the surface of the sample was calculated as *j × t*, where *j* is the flux of oxygen atoms to the surface and *t* is the treatment time. Various treatment times were used for modification of the sample’s surface. Such an experimental setup allowed for the treatment of samples in a broad range of fluences from 5 × 10^21^ to 3 × 10^24^ m^−2^—almost three orders of magnitude.

### 2.3. X-ray Photoelectron Spectroscopy (XPS) Characterization

Chemical composition of the samples was determined with an XPS instrument model TFA XPS (Physical Electronics, Ismaning, Germany) from Physical Electronics. Analyses were performed 15 min after the plasma treatment. Monochromatic Al Kα_1,2_ radiation at 1486.6 eV was used for sample excitation. Photoelectrons were detected at an angle of 45° with respect to the normal of the sample surface. XPS survey spectra were measured at a pass-energy of 187 eV using an energy step of 0.4 eV. High-resolution spectra of carbon C1s were measured at a pass-energy of 23.5 eV using an energy step of 0.1 eV. Because the samples are insulators, an electron gun was used for the additional charge compensation. The spectra were analyzed using MultiPak v8.1c software (Ulvac-Phi Inc., Kanagawa, Japan, 2006) from Physical Electronics.

### 2.4. Atomic Force Microscopy (AFM) Measurements

The surface morphology of the samples was analyzed with an AFM (Solver PRO, NT-MDT, Moscow, Russia). Images were recorded in a tapping mode using ATEC-NC-20 tips (Nano And More GmbH, Germany). A resonance frequency of the tip and the force constant were 210–490 kHz and 12–110 Nm^−1^, respectively. An average surface roughness of the samples (Ra) was determined by using the program Spip 5.1.3 (Image Metrology A/S). The average surface roughness was calculated from the images taken over an area of 5 × 5 µm^2^.

### 2.5. Contact Angle Measurements

Changes of the surface wettability of the plasma-treated samples were determined immediately after the plasma treatment. An instrument by See System (Advex Instruments, Brno, Czech Republic) was used. A demineralized water droplet of a volume of 3 μL was applied to the surface. The measured contact angles were analyzed by the software supplied by the producer. For each sample, three measurements were taken to minimize the statistical error.

## 3. Results and Discussion

**Figure 2:**
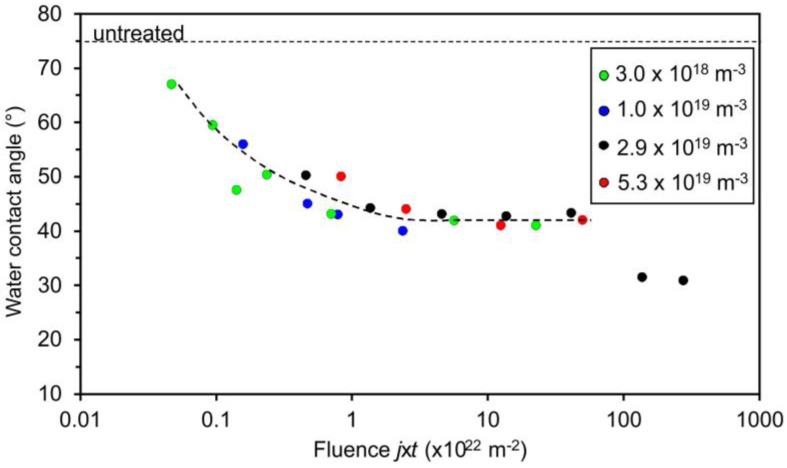
Variation of the water contact angle of the plasma-treated acryl-coated PP with the fluence of oxygen atoms. The different colors represent experiments with different O-atom densities.

Figure 2 illustrates the variation of the water contact angle of the acrylic coating versus the fluence of oxygen atoms. As mentioned earlier, the treatment was performed at several different densities of O atoms in the vicinity of the sample and at various treatment times. It seems that the water contact angle only depends on the fluence and not on the O-atom density because all measured points in Figure 2 follow the same curve. The contact angle at first decreases rapidly with the increasing fluence, but later the decrease becomes less and less rapid until the water contact angle becomes constant at approximately 40°. The particular measured points in Figure 2 are somehow scattered; however, the trend is obvious: no knee is observed in the curve which is only plotted for eye guidance. The contact angle becomes constant (approximately 40°) after the fluence of a few 10^22^ m^–2^ is used. Further exposure to O-atoms does not influence the wettability of this particular material. The exemptions are both measured points at very large fluences where the contact angles are approximately 30°. A feasible explanation for this effect will be presented and discussed later in this paper.

**Figure 3:**
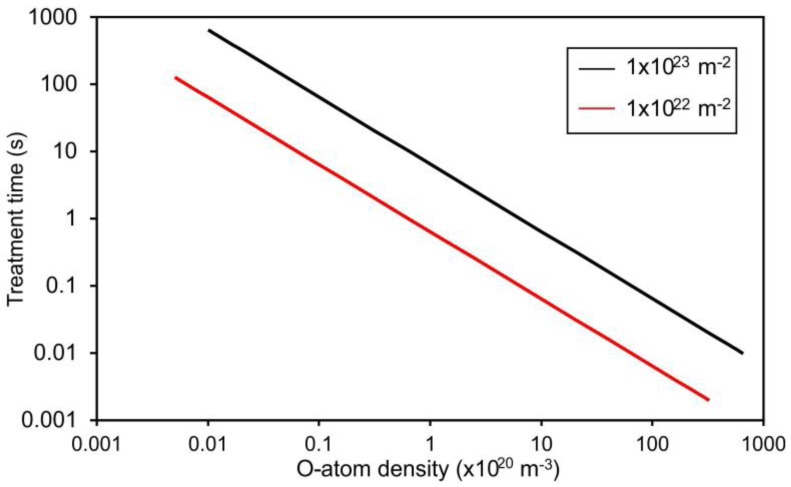
Recommended treatment times for achieving good wettability (~40°) of the acryl-coated polypropylene foils at two O-atom fluences.

Figure 3 represents the required treatment time for the fluences of 1 × 10^22^ and 1 × 10^23^ m^–2^. From this figure, one can conclude that the required treatment time for receiving the fluence of 1 × 10^22^ m^–2^ is only 6 ms at the atom density of 1 × 10^22^ m^–3^, which is typical for the extremely reactive oxygen plasma [39]. Such a short treatment time is achievable only when using pulsed discharges. Unfortunately, this experimental setup does not allow for verification of the calculated values presented in Figure 3. Furthermore, in practice, such small treatment times are not very suitable, because the treated surface may be contaminated with impurities. This means that at such a short treatment time, plasma radicals interact with the contaminants rather than with a pure polymer surface.

**Figure 4:**
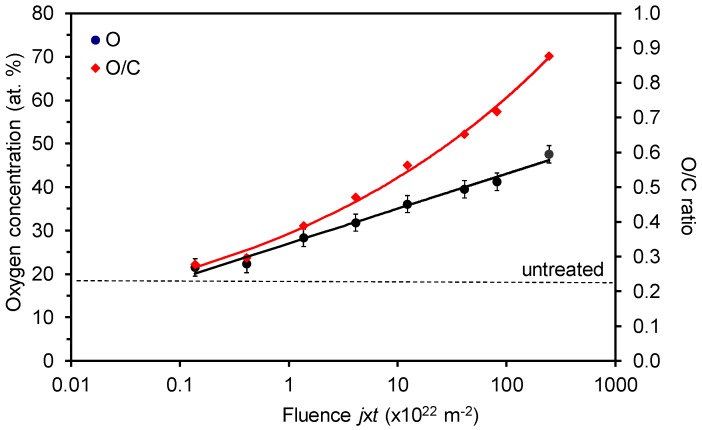
Variation of the oxygen concentration and the O/C ratio on the acryl-coated PP polymer surface with the O-atom fluence.

Figure 4 shows the variation of oxygen concentration and the O/C ratio on the polymer surface as determined by XPS. The oxygen concentration of the untreated sample was approximately 18 at %. The rather high concentration of oxygen in the surface film as detected by XPS (several nm thick) arises from the acrylic coating. After the treatment, the oxygen concentration on the surface increased. The increase is at first rapid but then less pronounced; however, the x-axis in Figure 4 is plotted in the logarithmic scale and therefore the measured points appear in a line. The oxygen concentration thus increases as a logarithm of the fluence. It is interesting that the oxygen concentration keeps increasing after the fluence that corresponds to the saturation of the wettability. Numerous explanations can be stated for this observation. A trivial one is that already approximately 30 at % of oxygen is enough for the optimal wettability. The second possibility is that the surface (which influences the wettability) is already saturated with the polar functional groups at a moderate fluence and oxidation of the sub-surface layers occurs at higher fluences. Yet another explanation could be the formation of oxides on the surface—this effect will be discussed later.

**Figure 5:**
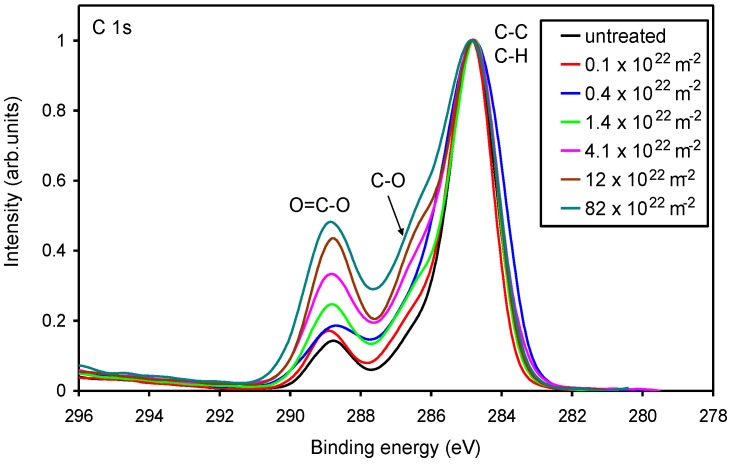
Comparison of high-resolution XPS carbon C 1s spectra of the acryl-coated PP polymer. The parameter is the O-atom fluence.

**Figure 6:**
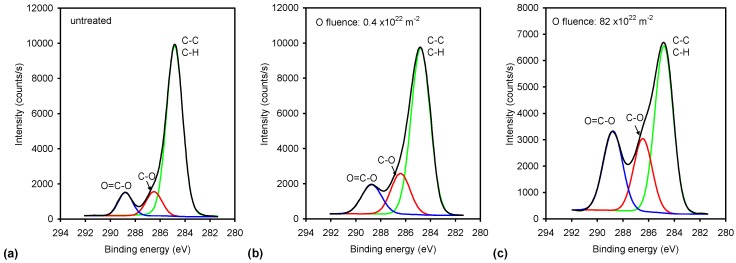
An example of fitting of XPS spectra: (**a**) untreated sample; (**b**) sample treated with a low O-atom fluence; and (**c**) sample treated with a high O-atom fluence.

**Figure 7:**
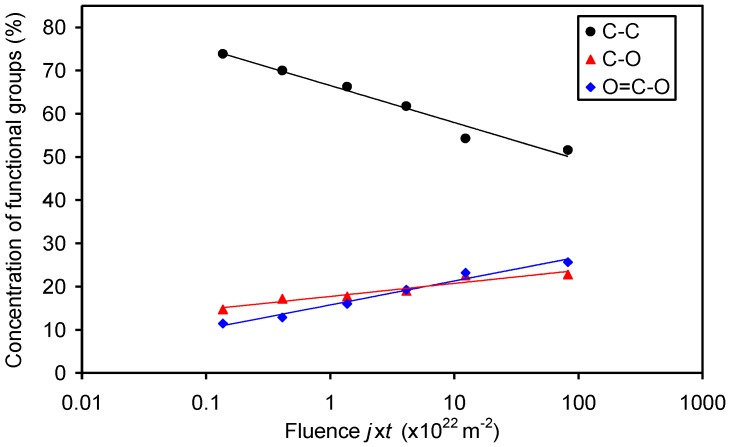
Variation of the concentration of various oxygen functional groups versus oxygen fluence. Concentrations were determined by fitting C 1s XPS spectra.

The high-resolution spectra of the carbon C1s peak for selected samples are presented in Figure 5. The spectra are normalized to the height of the main peak at 285 eV. The deconvolution of selected spectra is presented in Figure 6. The untreated sample (Figure 6a) contains three peaks: the main one at 285 eV corresponding to C–C, C–H bonds, and two small peaks at 286.5 and 289 eV corresponding to C–O and O=C–O groups, respectively. The spectrum in Figure 6a supports the information that the original sample has the acrylic coating. Figure 6b,c show an example of deconvolution of the sample treated at short (low oxygen fluence) and long (high oxygen fluence) treatment times. It can be observed that the intensity of C–O and O=C–O groups increased, especially for longer treatment times. It is difficult to judge about the formation of additional peaks corresponding to functional groups like C=O; however, if such groups develop upon treatment with the O atoms, their concentration on the polymer surface is much lower than the concentration of C–O and O=C–O groups. Figure 5 shows a gradual increase of the polar functional groups versus the fluence of the O-atoms, thus it is in good agreement with Figure 4. The increase is not equal for C–O and O=C–O groups, though. This can be seen from Figure 7, which shows the concentration of the functional groups versus the O-atom fluence. The highly polar O=C–O group increases somehow more intensively than the C–O group and actually prevails at the highest fluence. Interesting enough, this observation is not sound with the wettability presented in Figure 2. Namely, on the basis of the results presented in Figure 7, one would expect a monotonous decrease of the water contact angle with the increasing O-atom fluence. As mentioned above, this phenomenon could be related to surface saturation with the polar functional groups already at moderate fluences, and to oxidation of the sub-surface layers at higher fluences, or to the formation of Si oxides (discussed later).

**Figure 8:**
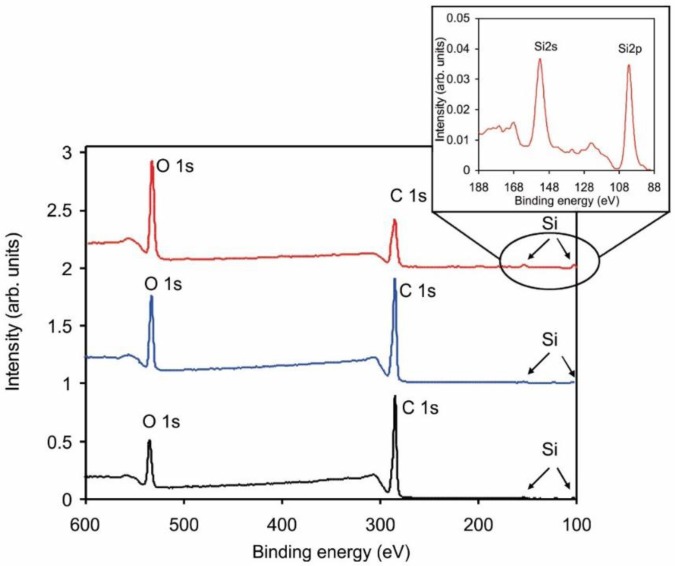
Selected XPS survey spectra of the untreated (**lowest curve**) and treated polymer at a low fluence of 0.4 × 10^22^ m^–2^ (**middle**) and at a high fluence of 82 × 10^22^ m^–2^ (**upper curve**).

**Figure 9:**
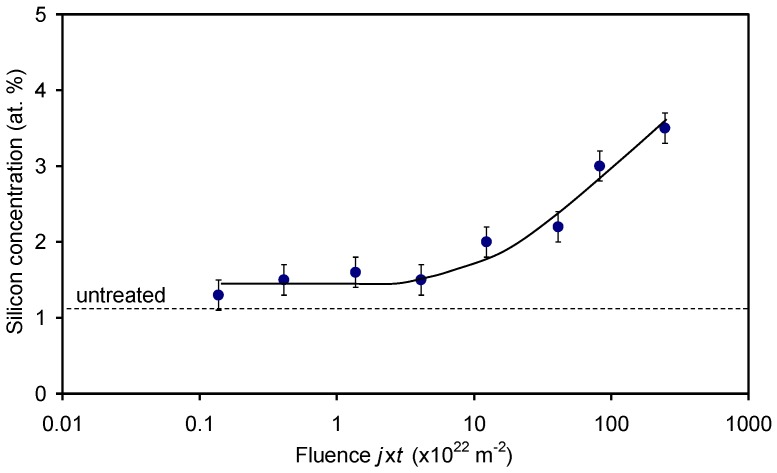
Silicon concentration versus O-atom fluence.

Another observation about the surface composition is worth stressing and discussing. Figure 8 represents survey XPS spectra for selected samples. Apart from carbon and oxygen, one can observe tiny peaks at binding energies of approximately 102 and 153 eV. The peaks correspond to silicon levels of Si 2p and Si 2s, respectively. The peaks are easily overlooked for the untreated sample (lowest curve in Figure 8), but become more pronounced after the sample has received a large fluence (upper curve). Detailed spectrum in the range 88–188 eV is shown in the insert of Figure 8. Doubtlessly, silicon is presented in the as-received sample and its concentration as detected by XPS increases with the increasing O-atom fluence. Figure 9 represents the concentration of Si in the surface of selected samples. Although the initial concentration is at the limit of this experimental technique, the trend is well justified. The origin of Si in the untreated sample is known to polymer scientists: i.e., silicon is often added to polymers as an anti-block or slipping agent in order to improve their performance. When the polymers are exposed to oxygen atoms, etching occurs. The effect has been elaborated elsewhere [40]. The oxygen atoms at first cause surface functionalization, but as the polymer surface becomes saturated with the O-rich functional groups, they form unstable molecular fragments which desorb from the surface. The polymer is thus slowly etched, leaving on the surface compounds that do not form volatile oxides. The effect is sometimes called plasma ashing [41]. Here, the acryl coating is slowly degraded and thus etched, leaving oxidized silicon nanoparticles on the surface. This effect explains the increase of Si concentration versus the O-atom fluence presented in Figure 9. It may or may not be a coincidence that the Si concentration (Figure 9) starts rising as the sample wettability becomes stable (Figure 2).

**Figure 10:**
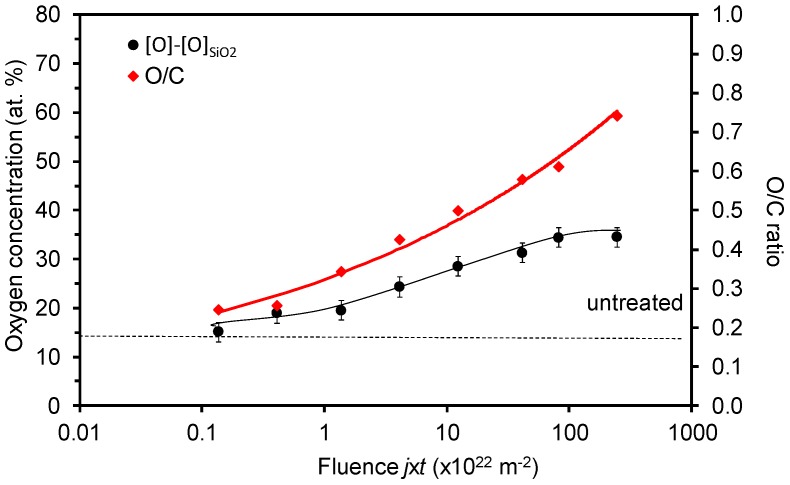
Variation of the oxygen concentration and the O/C ratio on the acryl-coated PP polymer surface with the O-atom fluence for the case when oxygen bonded to silicon is subtracted.

The virtual discrepancy between Figure 2 and Figure 4 can be attributed to the appearance of silicon on the polymer surface. As explained above, the wettability (Figure 2) assumes a rather constant value after the fluence of about 3 × 10^22^ m^−2^, but the concentration of oxygen on the polymer surface still increases (Figure 4). Taking into account the measured values of Si (Figure 9) and assuming that silicon is in the form of oxide (SiO_2_), one can replot Figure 4 by considering that a part of oxygen is bonded to silicon, i.e., subtracting 2 × [Si] oxygen from the curves. The new plot of O concentration and the O/C ratio by considering this effect is plotted in Figure 10. The behavior of the curve for oxygen in Figure 10 is now almost sound with the observations presented in Figure 2. Namely, the oxygen concentration as determined from XPS results also approaches a constant value for large fluences. Unfortunately, the saturation in Figure 10 does not appear at the same fluence as in Figure 2.

The role of silicon dioxide on the sample wettability is worth discussing. Figure 2 represents numerous measured data that fit the curve well, but the two points at the highest fluences definitely do not fit the general behavior. The decrease of the WCA for the highest fluences could be explained by oxidized silica nanoparticles on the sample surface, because well activated silicon oxide (treated by oxygen plasma) is hydrophilic [42]. The hydrophilicity is, however, lost soon after the plasma treatment because of the adsorption of organic impurities. That is one of the reasons why wettability tests were performed just after the treatment of samples with the O-atoms; however, hydrophobic recovery cannot be excluded completely.

**Figure 11:**
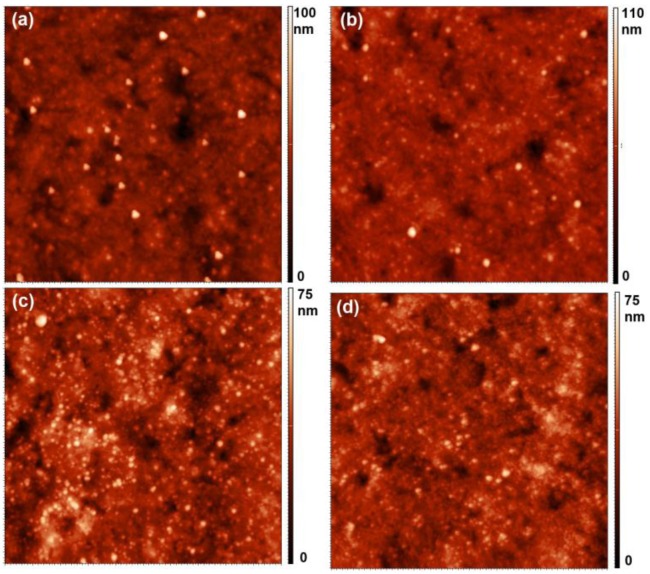
AFM images (5 × 5 µm^2^) of selected samples: untreated (**a**) and treated at various fluences: (**b**) 0.1 × 10^22^ m^–2^; (**c**) 82 × 10^22^ m^–2^; and (**d**) 247 × 10^22^ m^–2^.

In view of the upper discussion, let us also discuss the AFM images of selected samples. The images are shown in Figure 11. The images were taken over the area of 5 × 5 µm^2^. The untreated sample (Figure 11a) exhibits small un-evenly distributed particles of virtually the same lateral size protruding from the surface. The typical lateral dimension of the particles is almost 100 nm and the height as determined by AFM is several 10 nm. The origin of these particles is probably polymer additives containing silicon. According to the XPS results (Figure 9), the density of the particles fits the concentration of silicon on the surface of the untreated sample. Figure 11b is the image of the sample after receiving a small O-atom fluence. According to the upper results and discussion, the fluence received by this sample was too small to cause any detectable polymer etching. The image actually does not differ significantly from Figure 11a. Also, the surface roughness of the sample shown in Figure 11b did not change much (from the initial 5.8 nm it increased to 5.9 nm). One can qualitatively conclude that the concentration of the particles protruding from the sample surface is similar in Figure 11a,b, which is sound with the observations presented in Figure 9.

The AFM images in Figure 11c,d vary significantly from Figure 11a,b. The particles protruding from the surface are now much denser, which could be a consequence of the polymer etching. Moreover, the surface roughness increased to 6.8 nm. From Figure 11, one can therefore assume that the surface is enriched with silica nanoparticles, which has been proposed on the basis of the XPS results presented in Figure 9.

**Figure 12:**
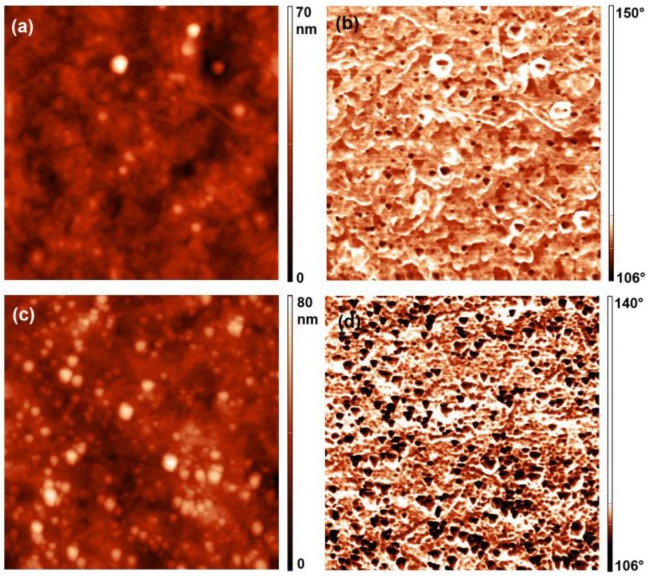
AFM topography (2 × 2 µm^2^) and phase images of selected samples: (**a**) topography of the untreated sample; (**b**) phase image of the untreated sample; (**c**) topography of the sample treated with a fluence of 82 × 10^22^ m^–2^; and (**d**) phase image of the sample treated with a fluence of 82 × 10^22^ m^–2^.

In Figure 12, AFM topographic and phase images of the untreated sample and of one the selected treated sample recorded at a higher magnification of 2 × 2 µm^2^ are shown. The phase signal depends on the viscoelastic properties of the materials; therefore, the signal variation between the soft polymer surface and stiff silica particles can be observed. Figure 12 clearly shows a big difference in the variation of the phase signal for the treated sample in comparison to the untreated one. Many black spots with a big phase shift are observed on the treated sample, which confirms our conclusions about the presence of silica particles.

## 4. Conclusions

An early stage of activation of commercial acrylic coated polypropylene foils for food packaging has been elaborated. The results clearly show that the maximum achievable surface wettability is already obtained at a rather low fluence of O-atoms of the order of a few 10^22^ m^–3^. This information is particularly useful for users who want to activate the material without losing the acrylic surface film. Namely, larger fluences (in practice it means prolonged treatment time) has little or no effect on the surface wettability but causes etching of the thin acrylic film and thus loss of the functional properties of such foils. As stated in the introduction to this paper, the acrylic coating protects the polypropylene foil from external influences, and should therefore remain on the PP foil after accomplishing the activation procedure.

## Figures and Tables

**Table 1:** Treatment conditions and results obtained by other authors.

Plasma Treatment	Discharge Parameters	Wettability	Surface Analysis	Reference
Low-pressure oxygen plasma	− RF source 8–12 MHz − Power: 7.2, 10.2, 29.6 W − Pressure: 26.7–80 Pa − Flow: 5–10 scfh − Exposure time: up to 5 min	WCA ^1^ was decreasing with increasing power and treatment time. From 74.5° it decreased to approx. 44° at the highest power of 29.6 W and at the longest treatment time 300 s. SFE ^2^ increased from 56.5 to max. 94.1 mJ/m^2^.	AFM ^3^: surface roughness RMS ^4^ increased from 1.5 to 7.3 nm. XRD ^5^: higher degree of crystallinity observed after oxygen plasma treatment. Mechanical properties: tensile strength decreased after plasma treatment. Barrier properties: water vapor transmission was increasing with increasing power and time.	[24]
Low-pressure oxygen and argon plasma	− commercial plasma reactor from Diener Co. − LF plasma 40 kHz − Power: 50 W − Pressure: 0.2 × 10^5^ Pa − Flow: 50 cm^3^/min − Exposure time: up to 5 min	Increase of the surface energy of oxygen plasma-treated sample was higher than for the one treated in Ar. SFE was 70 and over 50 mJ/m^2^ for O_2_ and Ar plasma, respectively.	ATR-FTIR ^6^: carbonyl groups observed as well as C=C which could be a sign of crosslinking. AFM: O_2_ plasma caused higher roughness than Ar. Adhesion: High improvement of surface adhesion strength, especially for O_2_ plasma.	[32]
Low-pressure oxygen plasma	− RF source 13.56 MHz, capacitive − sample placed on the grounded electrode − Power: 70 W − Pressure: 6.7 Pa − Flow: 49 sccm − Exposure time: up to 5 min	WCA decreased from 110° to 40°.	AFM: surface roughness first decreased with treatment time. At longer treatment times, a significant increase is observed. FTIR: C=O and –OH peaks observed for plasma-treated samples.	[23]
Low-pressure air plasma	− DBD plasma − sample placed on the grounded electrode − AC power source 10 kHz − Power: 1.4 W − Pressure: 5 × 10^3^ Pa − Flow: 20 sccm − Exposure time: 0.2–30 s − Energy load: up to 3.34 J/cm^2^	WCA decreased from 94.9° to 60°. Saturation reached after 10 s.	XPS ^7^: Oxygen concentration increased from 4.3 to 13.7 at %. Nitrogen (0.8 at. %) was also found, the rest being carbon. 8.2% C–O, 2.7% C=O and O–C=O and 86.4% C–C, C–H groups observed on plasma treated sample. ATR-FTIR: peaks attributed to OH and C=O in ketones, aldehydes and carboxylic acids.	[21]
Atmospheric pressure air plasma	− DBD plasma − “Coating Star” device from Ahlbrandt System − sample placed on the grounded electrode − 30 kHz, 15 kV − Power: 300–1000 W − Pressure: atmospheric − Flow: 20 sccm − Treatment speed: 2–10 m/min − Energy load: up to 60 kJ/m^2^	WCA decreased from 104° to 64° even at 1.2 kJ/m^2^. SFE increased from 33.7 to almost 50 mN/m.	XPS: O/C ratio increased over 0.16. Nitrogen (2 at %) was also found. After one month, O/C decreased to 0.12. Groups like C–O (22.5%), C=O or O–C–O (8.4%) and O=C–O (5.3%) were found. Maximum concentration was obtained at the lowest treatment speed. AFM: Ra ^8^ increased from 5.8 to 12.9 nm. Bumps were observed on the surface. The height and width were increasing with treatment power and reached 60 and 500 nm, respectively.	[22]
Low-pressure oxygen and argon plasma	− RF source 13.56 MHz − Commercial K 1050X Plasma Asher Model from Emitech Co. − sample placed in the middle of the chamber on the glass substrate − Power: 10, 30, 50 W − Pressure: 0.35 × 10^5^ Pa − Flow: 15 mL/min − Exposure time: up to 5 min	WCA was decreasing with increasing power and treatment time. The lowest WCA was 34.4° for O_2_ and 38.2° for Ar plasma (initially 98.3°). SFE increased to ~45 mN/m.	SEM ^9^ and AFM: topology and roughness changed significantly, especially for Ar plasma (nodules observed on the surface). RMS roughness increased from 3.6 to 6.9 and 6.1 nm for O_2_ and Ar plasma, respectively. ATR-FTIR: C=O stretching bond and C=C vibration observed. Some peaks attributed also to carboxylic/ester, aldehydes and ketone groups.	[25]
Low-pressure oxygen plasma	− RF power source − sample was on the tray in the middle of the chamber − Power: 500 W − Pressure: 13.3 Pa − Flow: 49 sccm − Exposure time: up to 40 min	WCA decreased from 121.5° to 84° on PP nonwoven mats. Ageing for 90 days did not have significant effect on WCA. SFE increased from 13.7 to 29.2 mN/m.	SEM: etching of PP fibers observed. XRD: no significant effect on the crystallinity of the treated fibers.	[33]
Low-pressure oxygen plasma	− RF source 13.56 MHz, capacitive − Power: 0–150 W − Pressure: 0–120 Pa − Exposure time: 30 s–3 min − Ageing: 30 days	The lowest WCA—bellow 10° was observed at 150 W, 3.33 Pa and 60 s. After 30 days of ageing WCA increased to ~50°.	Ageing and crystallinity: Two polymers with different initial crystallinity were used. More crystalline PP was ageing slower—WCA after 30 days was for ~5° lower than for less crystalline one. Degree of crosslinking was increased after the treatment for both polymers. XPS: ~25 at % of oxygen was found on less-crystalline polymer. O concentration on more crystalline polymer was few at % lower. However, after ageing the O concentration changed in favor of more crystalline one.	[34]
Low-pressure oxygen plasma	− RF power source − commercial reactor Inverse Sputter Etcher ISE 90 model 2001 (Von Ardenne Anlagentechnik − GmBh) − Power: 50 W − Pressure: 5.1 Pa − Exposure time: up to 40 min	WCA decreased from 98° to 24°. At long treatment times, it increased to 53°.	AFM: roughness RMS increased from ~ 12 nm to ~44 nm. ATR-FTIR: OH, C=O and CO–C=O peaks observed for plasma treated samples.	[26]
Low-pressure oxygen plasma	− DC plasma (20 mA, 2 kV) − sample was placed on glass walls of discharge chamber positioned between the electrodes separated 42 cm − Pressure: 30 Pa − Exposure time: up to 200 s	WCA decreased from 83° to 60°. SFE increased from 25.7 to 43 mJ/m^2^.	ATR-FTIR: OH, C=O groups in ester, ketone and carboxyl groups, C=O groups in unsaturated ketones and aldehydes.	[20]
Low-pressure oxygen plasma	− Capacitor plate plasma − commercial K1050 X Plasma Asher from Emitech Ltd. − sample positioned on the holder − Power: 0–100 W − Pressure: 60 Pa − Flow: 15 mL/min − Exposure time: up to 10 min − Ageing: 90 days in air or water	WCA was decreasing with the increasing power and treatment time. Minimal achievable WCA was 55.6° (initially 103°). Ageing in water was faster than in air. After 90 days, the WCA was 81.7° (in water) and 71.2° (in air).	AFM: Roughness RMS increased after treatment from 2.1 nm to ~10 nm (in air) and ~5 nm (in water). Lower roughness of samples stored in water was explained by removing of water-soluble short-chain species.	[35]

^1^ Water contact angle (WCA); ^2^ Total surface free energy (SFE); ^3^ Atomic-force microscopy (AFM); ^4^ Root mean squared (RMS) roughness; ^5^ X-ray diffraction (XRD); ^6^ Attenuated total reflection Fourier transform-infrared spectroscopy (ATR-FTIR); ^7^ X-ray photoelectron spectroscopy (XPS); ^8^ Average roughness (Ra); ^9^ Scanning electron microscope (SEM).
